# Fortified whey beverage for improving muscle mass in chronic obstructive pulmonary disease: a single-blind, randomized clinical trial

**DOI:** 10.1186/s12931-020-01466-1

**Published:** 2020-08-17

**Authors:** Afsane Ahmadi, Mohammad Hassan Eftekhari, Zohreh Mazloom, Masoom Masoompour, Mohammad Fararooei, Mohammad Hadi Eskandari, Samrad Mehrabi, Alireza Bedeltavana, Mandana Famouri, Morteza Zare, Nasrin Nasimi, Zahra Sohrabi

**Affiliations:** 1 Research Center for Health Sciences, Institute of Health, Shiraz University of Medical Sciences, Shiraz, Iran; 2 Department of Clinical Nutrition, School of Nutrition and Food Sciences, Shiraz University of Medical Sciences, Shiraz, Iran; 3 Non-communicable Disease Research Center, Shiraz University of Medical Sciences, Shiraz, Iran; 4 Department of Epidemiology, School of Health, Shiraz University of Medical Sciences, Shiraz, Iran; 5 Department of Food Sciences and Technology, School of Agriculture, Shiraz University, Shiraz, Iran; 6 Department of Internal Medicine, School of Medicine, Shiraz University of Medical Sciences, Shiraz, Iran; 7 Dairy Expert at Research and Development of Zarrin Ghazal Company (DAITY), Shiraz, Iran; 8 Nutrition Reasearch Center, School of Nutrition and Food Sciences, Shiraz University of Medical Sciences, Shiraz, Iran; 9 Department of Community Nutrition, School of Nutrition and Food Sciences, Shiraz University of Medical Sciences, Shiraz, Iran

**Keywords:** Lung diseases, Whey, Magnesium, Vitamin C, Cachexia

## Abstract

**Background:**

The development of effective nutritional supports for patients with chronic obstructive pulmonary diseases (COPD) is still challenging. This study was conducted to investigate the efficacy of daily consumption of fortified whey on inflammation, muscle mass, functionality, and quality of life in patients with moderate-to-severe COPD.

**Methods:**

A single-blind, randomized trial study was performed on patients with COPD (*n* = 46). Participants in the intervention group (*n* = 23) daily received 250 ml of whey beverage fortified with magnesium and vitamin C for 8 weeks. Any changes in inflammatory cytokines (including interleukin- 6 (IL-6) and tumor necrosis factor (TNFα)) were the primary outcomes and the secondary outcomes were fat-free mass, handgrip strength, malnutrition, glutathione and malondialdehyde serum concentrations, and health-related quality of life (HRQoL). Body composition and muscle strength were measured by Bioelectrical Impedance Analysis (BIA) and hydraulic hand dynamometer, respectively. Fat-free mass index (FFMI) was also calculated.

**Results:**

At the end of the study, 44 patients were analyzed. There were significant decreases in IL-6 concentrations in the intervention group compared to the control group. Also, FFMI, body protein, and handgrip strength increased significantly in the intervention group with significant changes between two groups. Moreover, improvement in health-related quality of life was observed in the intervention group compared to the control group. There were no significant changes in other study variables.

**Conclusions:**

This novel nutritional intervention decreased inflammatory cytokines levels, improved indices of skeletal muscle mass and muscle strength, and ultimately, increased HRQoL in patients with moderate-to-severe COPD. Thus, it is suggested to do further studies to assess the effects of nutrition intervention on COPD progression.

**Trial registration:**

IR.SUMS.REC.1396.85 (https://www.irct.ir/).

## Background

Chronic obstructive pulmonary disease (COPD) is a respiratory disorder characterized by progressive airflow obstruction and persistent lung inflammation [1]. COPD has been a major public health concern, due to its high prevalence and adverse health consequences, such as worsening dyspnea, functional disability, hospitalization, poor quality of life, and increased mortality [2]. Also, COPD-related mortality rate is expected to be the third leading cause of death worldwide in 2030 [3]. This disease is a preventable disorder, which smoking and aging are its main contributing factors [4]. COPD progression causes a great economic and social burden, therefore, early detection and effective treatment is essential [5].

Muscle wasting and unintentional weight loss are common as the COPD extra-pulmonary pathology, which might lead to peripheral and respiratory muscle dysfunction, reduced exercise capacity, decreased independence, and increased mortality [6]. The underlying mechanisms of muscle wasting and dysfunction are multifactorial in COPD. Malnutrition, chronic systemic inflammation, and smoking-induced oxidative stress are the major prominent related factors [7]. COPD has been introduced as a new syndrome called “chronic systemic inflammatory syndrome” associating with many comorbidities and metabolic consequences [8].

Nutritional support is already a major therapeutic strategy in preventing disease progression and complications [9]. Based on the available evidence, dietary whey protein and magnesium are considered as good candidates regarding the role of particular nutrients in muscle preservation and inflammation reduction, and due to their specific metabolic and anti-inflammatory properties. Whey protein is considered as a high-quality dietary protein with high content of branched chain amino-acids. This protein might help stimulating muscle synthesis through providing essential amino acids and enhancing protein anabolism [10]. Whey protein also has antioxidant and anti-inflammatory properties, which might be effective in reducing the circulation of pro-inflammatory cytokines, such as interlukine-6 (IL-6) and tumor necrosis factor (TNF-α) [11].

Magnesium is another nutrient with a great importance in COPD. Magnesium is considered as a disease severity predictor in patients with COPD. Recent evidence has shown that magnesium levels in COPD are decreased, and inadequate dietary magnesium intake might lead to reduction in skeletal muscle mass, strength, and power, as well as COPD progression [12]. Muscle mass might be preserved by dietary magnesium due to its direct effects on physiological mechanisms (such as protein synthesis, oxygen uptake, fat oxidation, and electrolyte balance), as well as reducing the systemic inflammation and pro-inflammatory cytokine levels [13].

Moreover, high oxidative stress in COPD might cause more muscle degeneration and worsen other symptoms. Inflammatory mediators’ expression by reactive oxygen species might lead to muscle cell deterioration through actin and myosin apoptosis and damage [14, 15]. Recent studies suggested that there might be a relationship between dietary intake of antioxidants, such as vitamin C, and improved lung function, reduced lung infection, and COPD-related symptom and severity [16].

A multi-ingredient nutritional intervention, including whey beverage fortified with magnesium and vitamin C, was selected according to the reduced skeletal muscle mass, chronic systemic inflammation, and impaired antioxidant capacity in patients with COPD, as well as the role of nutrition support in improving these symptoms. Given the metabolic and anti-inflammatory effects of whey protein and magnesium, as well as the antioxidant effects of vitamin C and whey protein, we was aimed to evaluate the effect of the combination of these nutrients, independent of exercise training on muscle mass maintenance and quality of life in patient with moderate-to severe COPD,

## Methods

### Study design

The efficacy of whey beverage fortified with magnesium and vitamin C on patients with COPD was evaluated in this single-blind, randomized controlled trial. The study protocol was approved by the Ethics Committee of Shiraz University of Medical Sciences, Iran, and it was performed in accordance with good clinical practice and the Declaration of Helsinki. The protocol is available at www.irct.ir, with identification cod IR.SUMS.REC.1396.85, registered 29 December 2018. Before the initiation, the study purpose and procedure were described for all of the eligible patients, and a written consent form was completed. Data were collected from September 2018 to February 2019 (intervention duration from January to February 2019).

### Participants

The study population consisted of male patients with clinical diagnosis of moderate-to-severe COPD, whose medical information were recorded at four main educational hospitals affiliated to Shiraz University of Medical Sciences (SUMS) (Nemazee, Rajayi, Ali-asghar, and Shahid Faghihi hospitals) and specialized respiratory clinics between March 2011 and June 2018. The participants meeting inclusion criteria were male patients aged 50–70 years, with moderate-to-severe COPD (according to spirometry test and FEV1 index), diagnosed with moderate COPD for at least 6 months, with BMI < 25 kg/m^2^, with fixed dose and type of medications, not being hospitalized in the last 3 months, and living in Shiraz. In addition, patients with a history of cardiac failure, malignancy, severe renal, hepatic or rheumatic disorders, and active inflammatory or infection were not enrolled. Moreover, the other main exclusion criteria was regular use of antioxidants, mineral or protein supplements (vitamin C, magnesium, etc.). During the study phase, participants were excluded if they refrained to drink fortified whey beverage and experienced serious side effects and hospitalization due to increased illness severity.

Sample size was assigned based on interleukin-6 concentration (as a primary outcome), from the published study (by considering α = 0.05 and power = 80%) [17]. Accordingly, the final sample size in each of the intervention and control group was calculated to be 23 patients including 15% drop-out rate.

### Intervention

In this study, 147 eligible participants were ultimately screened out of 1957 patients who were monitored and ultimately 46 patients were eligible for participation. In the first phases, identical COPD nutrition booklets were given to all participants. The booklet provided comprehensive information on nutrition requirements (with emphasis on nutrients such as protein, magnesium, vitamins, etc.), nutrition problems, and coping strategies. Moreover, individual nutritional counseling was also considered for all patients.

After a run-in period, participants were randomly divided into two intervention and control groups in a 1:1 ratio, using balanced block randomization (BBR). Also, the allocation was stratified based on BMI (< 23 and > 23) [18] and disease severity (predicted FEV1 < 50 and predicted FEV1 > 50) [19]. The randomization procedure with a block size of 4 was performed by an independent statistician.

Participants in the intervention group daily received 250 ml of whey beverage fortified with magnesium and vitamin C (each 250 ml contained 275 mg elemental magnesium, 685 mg vitamin C, and 15.9 g whey protein) with dietary advice and routine care for 8 weeks. Participants in the control group just received dietary advice and routine care. Participants in the intervention group were informed by telephone and were given fortified whey beverages at their homes. Patients in the intervention and control groups had no information contaminations during the study period.

The whey beverage fortified with magnesium and vitamin C was weekly manufactured by Zarin Ghazal Dairy Industries Company (DAITY, Shiraz, Iran). This fortified beverage was provided in ready-to-drink and non-transparent bottles of 1750 ml, which every 250 ml contained 15.9 g whey protein concentration (NCB-Laan 80; DMV International., Netherlands), 625 mg magnesium oxide (Product code: XM520184; HSH Chemical GmbH, Poland), 685 mg vitamin C (Product code: DY055150201; Northeast Pharmaceutical Group Co., Ltd., China), and with 113.6 Kcal energy (Table 1). Furthermore, suitable sweetener and flavor were added to the mixture to increase the product acceptance after various experimental productions, then pasteurization and homogenization were performed in standardized processing procedures. Moreover, all microbial and sensory analysis were weekly performed in order to ensure the safety and quality of the product throughout the study period.

**Table 1:** Nutrient composition of the nutritional beverage, per serving (250 ml)

Components	Fortified whey beverage
Energy, Kcal	113.6
Whey protein, g	15.9
Magnesium oxide, mg	625
Elemental magnesium, mg	275
Vitamin C, mg	685
Sugar, g	12.5

The stability of vitamin C in the product was determined using High Performance liquid chromatography (HPLC) technique during the 1 week refrigerated storage. Based on the results, vitamin C content in the product was calculated and it was determined that participants daily received an average of 500 mg of vitamin C, during the study. Moreover, whey protein and magnesium concentrations were measured using Kjeldahl (PecoTech) and atomic absorption (GBC AA32) methods, respectively. Reports showed that the concentration of whey protein in the product was constant during the 7 days of storage.

Participants were asked to record their daily drinking intake in the checklist and return the bottles at their weekly visits in order to evaluate their adherence. Participants’ compliance was checked based on the amount consumed by each of them. During the follow-up, both groups of participants received dietary advice through COPD nutritional booklets and weekly phone calls. Furthermore, any adverse events were recorded in all weekly meetings.

### Outcome measures

Inflammatory biomarkers were the primary outcome measures. The secondary outcome measures included any changes in malnutrition status, anthropometric variables, fat-free mass, muscle strength, and health-related quality of life (HRQoL).

### Anthropometrics data and body composition

Body weight and height were measured using a calibrated scale (Omron, Korea) and a portable stadiometer during which participants were wearing light clothes and their shoes were removed. Body mass index (BMI) was calculated by dividing body weight (kg) upon height squared (m^2^). Calf circumference was also measured using standardized techniques in seated position.

Body composition, including segmental lean body mass, fat-free mass (FFM), and fat mass were evaluated using a segmental multi-frequency Bioelectrical Impedance Analysis (BIA) and InBody S10 analyzer (BioSpace Co., Ltd., South Korea). The measured FFM was standardized for height squared (m^2^) in the form of fat-free mass index (FFMI).

### Strength assessment

Handgrip strength was measured using hydraulic hand dynamometer (model MSD, Sihan, Korea). The mean of three consecutive attempts for each hand was recorded.

### Lung function

The post-bronchodilator lung function parameters (including forced expiratory volume in 1 s (FEV1), forced vital capacity (FVC), and static lung volumes) was determined at screening, baseline, and week 8 using spirometry test (Vitalograph Pneumotrac 77,000 PC based Spirometry, England). All measured values were compared to normal values predicted by the European Community for Coal and Steel and European Respiratory Society, and they were expressed as percentages of the predicted values. FEV1 was defined as a disease severity indicator, which the COPD severe and moderate severity was presented by the value below 80% [20]. The highest value for FEV1 from 3 consecutive measurements was considered as the main value.

### Clinical assessment

The patients’ nutritional status was evaluated using a patient-generated subjective global assessment (PG-SGA) questionnaire for monitoring malnutrition. Also, nutritional history (including body weight changes, food intake, gastrointestinal symptoms, functional capacity, and respiratory stress) and physical examination were evaluated using this questionnaire. At the end, a score was given to any patient for showing the malnutrition degree according to different parts [21].

### Dietary assessment

Dietary intake was evaluated using a self-reported 3-day food record (2 weekdays and 1 weekend) at the baseline and after 8 weeks. Participants were instructed to report food quantities and ingredient, cooking methods, and volume and type of consumed liquids. Moreover, Energy and nutrient contents were calculated using the Nutritional IV software modified for Iranian foods (First Databank, San Bruno, CA, USA).

### Health-related quality of life (HRQoL) assessment

according to patients’ respiratory function, their HRQoL status were evaluated using St. George’s respiratory questionnaire (SGRQ), instrumental activities of daily living scales (IADLs), and Katz Index of Independence in Activities of Daily Living Scale.

The SGRQ is a comprehensive questionnaire used for monitoring HRQoL status and well-being in patients with COPD, and includes 14 items in 3 categories of symptoms (frequency and severity of respiratory symptoms), activity (role limitations due to breathlessness), and impact (psychological and social functioning disturbances due to respiratory disorder). The total score was calculated based on the sum score of each category in a range of 0–100, which the higher score indicated worse health status and more impairment [22].

Patients’ ability to perform living skills independently was evaluated using IADL scales. The total score was obtained from the sum of 8 domains ranging from 0 (severe functional impairment) to 8 (complete function). Moreover, patients’ dependency status in activities of daily living (ADL) was evaluated using Katz-ADL index. In this scale, participants were scored 0 (severely dependent) to 6 (independent), based on their performance in the 6 basic activities of bathing, dressing, toileting, transferring, continence, and feeding [23].

### Biochemical indicators

Blood samples were drawn from each participant before and after the intervention period. Serum samples were derived after centrifugation and were immediately stored at − 70 °C until analysis. Also, serum albumin concentrations were measured by an enzymatic method (commercial kit from Pars Azmun, Iran) using Auto-chemistry analyzer (BT1500; Biotecnica Instrument, Italy). Furthermore, plasma concentrations of Glutathione (GSH) were determined using color metrically kit (ZellBio GmbH, Germany) in order to evaluate antioxidant status.

Plasma inflammatory profiles, including tumor necrosis factor (TNFα) and interleukin-6 (IL-6), were determined by an enzyme linked immune sorbent assay (ELISA) method, using Demeditec and IBL international kits, respectively. The levels of serum malondialdehyde (MDA) were measured by modified thiobarbituric acid method, using spectrophotometric assays.

According to the standard protocol, plasma vitamin C concentrations were analyzed using a high pressure liquid chromatography (HPLC) system (Waters pump Binary 1525, USA). Blood samples were taken immediately from all participants in the intervention group after the end of the week 8 due to the rapid removal of vitamin C from the circulation, in order to evaluate vitamin C absorption. Furthermore, serum magnesium levels were measured by photometric assays, using a commercially available kit (Ziest CHEN, Iran).

### Statistical analysis

The results are displayed as mean ± standard deviation (SD) and the numbers (percentages) of continuous and categorical data, respectively. Shapiro-wilk test was used for all variables to evaluate normality distribution. The baseline characteristics between the intervention and control groups were compared using independent *t*-test or Mann-Whitney U test for the normally-distributed or skewed data, respectively. Also, paired t-test or Wilcoxon rank-sum test were used by considering variable normality in order to evaluate the changes in each group during the study phase. Moreover, the differences between both groups were analyzed using ANCOVA. As there were significant differences in dietary intake for protein and magnesium at baseline and 8th week of intervention, protein and magnesium were also considered as covariates. Data were analyzed using IBM SPSS Statistics, version 21, and *P* < 0.05 was considered statistically significant.

## Results

**Fig. 1:**
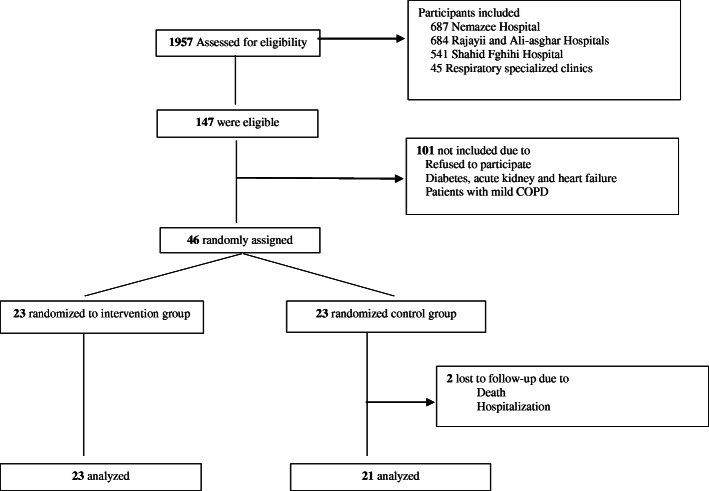
Flow diagram of participants screening, randomization and follow-up

In this study, 46 male patients with moderate-to-severe COPD were randomly assigned to the intervention or control groups, from whom 44 patients completed 8 weeks of intervention. Figure 1 indicates the patients’ flow diagrams through the study. Two participants were excluded from the control group due to death and hospitalization. The adherence of consumption of beverage fortified with magnesium and vitamin C showed that 18 (75%) participants in the intervention group had more than 90% adherence, and only 1 (4.2%) participant had less than 70% adherence during the study. Moreover, consuming the fortified whey beverage during the study phase showed no side effect in COPD patients except for small gastrointestinal symptoms including bloating in 2 patients and its consumption seemed to be safe in this group.

Baseline characteristics and measurements of the participants are shown in Table 2. Almost all of the participants (95.65%) had smoking habits, more than half were current smokers (56.52%), and the majority smoked for more than 20 years (75.6%). The patients were on average 62.76 years of age, were characterized by low FEV1 (mean 43.74%) and BMI (mean 21.08). At the baseline, mean fat-free mass and body protein were significantly higher in the intervention group, while there was no difference in other body composition and anthropometric measures between two groups. Also, there was no significant differences in mean dietary intakes of energy, protein, vitamin C, and Magnesium between the two groups. However plasma vitamin C concentration tended to be higher in the intervention group, with significant differences between the intervention and control groups. Moreover, although here were no significant differences in baseline SGA, IADL, and Katz variables between the intervention and control groups, the average plasma GSH concentration was lower in the intervention group. In addition, there were significant differences in SGRQ parameters between the two groups at baseline (Table 2). All of the baseline values that were statistically significant were adjusted as the covariate in ANCONA analysis.

**Table 2:** Baseline characteristics and measured parameters of the study participants

Variables	Control n = 23	Intervention *n* = 23	*P*-value
Age, yr.	63.47 ± 7.24	62.08 ± 7.0	0.506
**Smoking status**			
Smoking habit, yr.	33.26 ± 12.36	32.73 ± 14.89	0.902
Age starting smoking, yr.	25.22 ± 11.55	26.36 ± 10.65	0.693
**Body composition**			
Weight, kg	59.16 ± 7.66	59.92 ± 9.88	0.770
BMI, kg/m2	21.53 ± 2.59	20.65 ± 3.49	0.332
Calf circumference, cm	31.50 ± 2.01	31.41 ± 2.98	0.912
Arm circumference, cm	28.96 ± 2.66	28.60 ± 3.01	0.666
Lean body mass, kg	6.72 ± 0.46	6.98 ± 0.81	0.178
Protein BIA, kg	8.91 ± 0.97	9.64 ± 1.27	**0.035**
Fat-free mass, kg	45.35 ± 4.94	48.87 ± 6.17	**0.037**
FFMI, kg/m2	16.41 ± 0.89	16.79 ± 1.89	0.381
**Muscle strength**			
Right HGS, kg	23.35 ± 6.61	23.77 ± 6.58	0.709*
Left HGS, kg	21.7 ± 7.33	23.38 ± 8.24	0.376*
**Diet**			
Energy intake, kcal	1670.7 ± 656.9	1731.0 ± 689.7	0.947*
Protein intake, g	67.72 ± 30.15	55.55 ± 28.03	0.147*
Fat intake, g	45.41 ± 29.67	49.53 ± 33.01	0.758*
Carbohydrate intake, g	253.36 ± 113.61	268.30 ± 108.99	0.649
Vitamin C intake, mg	108.98 ± 122.86	125.71 ± 93.22	0.322*
Magnesium intake, mg	237.25 ± 116.54	182.06 ± 75.49	0.124*
Water, cup	1.37 ± 1.48	2.17 ± 2.60	0.447*
**Blood analysis**			
IL-6, pg/ml	11.67 ± 10.84	8.15 ± 10.16	0.057*
TNFα, pg/ml	30.04 ± 9.36	25.57 ± 10.42	0.135
GSH, mM/l	202.72 ± 40.66	176.7 ± 34.6	**0.024**
MDA, μM/L	2.90 ± 0.47	2.81 ± 0.48	0.544
Albumin, g/dl	4.15 ± 0.37	4.15 ± 0.27	0.949
Vitamin C, μM/L	27.00 ± 3.30	29.45 ± 4.47	**0.042**
Magnesium, mg/dl	1.98 ± 0.16	2.02 ± 0.16	0.323*
**Lung function**			
FEV1, %	44.95 ± 14.16	42.58 ± 16.74	0.604
FVC, %	60.54 ± 10.34	59.29 ± 10.83	0.636
SGA score	10.43 ± 4.81	10.37 ± 3.25	0.960
SGRQ, total score	59.0 ± 14.0	67.0 ± 13.0	**0.046**
SGRQ, symptom score	63.0 ± 18.0	73.0 ± 10.0	**0.025**
SGRQ, activity score	65.0 ± 20.0	71.0 ± 19.0	0.344
SQRQ, impact score	53.0 ± 16.0	63.0 ± 14.0	**0.037**
IADL score	5.04 ± 1.39	5.50 ± 0.93	0.231*
Katz index	5.56 ± 0.89	5.45 ± 0.93	0.459*

Presented values are mean ± SD.

^*^ Non-parametric (Mann-Whitney) test were used for variables

Abbreviations: *BMI* Body mass index, *FFMI* Fat-free mass index, *HGS* Handgrip strength, *GSH* Glutathione, *TNFα* Tumor necrosis factor, *IL-6* Interlukin-6, *MDA* Malondialdehyde, *FEV1* Forced expiratory volume in 1 s, *SGA* Subjective global assessment, *SGRQ* St. George’s respiratory questionnaire, *IADL* Instrumental activities of daily living

Changes in dietary intakes are shown in Table 3. There were a significant increases in protein and magnesium intakes (excluding fortified whey beverage) over the time in the intervention group compared to the control group (*P* = 0.021 and *P* = 0.005, respectively). Therefore, these two variables were adjusted as fixed factors in ANCOVA analysis for all measures due to their effects on the results (Table 3).

**Table 3:** Changes in dietary intake in patients after 8 weeks

Variables	Control group (*n* = 21)	Intervention group (n = 23)	
	change mean ± SD	change mean ± SD	P^1^
Energy intake, kcal	− 107.88 ± 677.34	49.04 ± 843.50	0.262^*^
Protein intake, g	−4.88 ± 34.62	21.22 ± 39.04	**0.021**
Fat intake, g	−5.7 ± 44.27	−0.92 ± 41.03	0.495^*^
Carbohydrate intake, g	−11.66 ± 128.86	−2.28 ± 119.21	0.799
Vitamin C intake, mg	−18.42 ± 147.11	−2.04 ± 155.54	0.982^*^
Magnesium intake, mg	−33.45 ± 125.96	82.27 ± 132.14	**0.005**
Water, cup	0.70 ± 1.65	0.52 ± 1.08	0.897^*^

^1^ *P* values denote significance of between-group changes

^*^ Non-parametric (Mann-Whitney) test were used for variables

Changes in outcome measurements are indicated in Table 4. Inflammatory biomarkers (including TNFα and IL-6) significantly decreased in the intervention group compared to the baseline during the study phase. However, there were significant differences between the two groups only in IL-6 levels (*P* = 0.034). Furthermore, plasma GSH concentration, as an antioxidant capacity biomarker, significantly increased in the intervention group.

**Table 4:** Effect of intervention on levels of measured parameters in COPD patients after 8 weeks

Variables	Control group (n = 21)				Intervention group (n = 23)				Intervention effect
	Pre mean ± SD	Post mean ± SD	Change mean ± SD	Intra P^1^	Pre mean ± SD	Post mean ± SD	Change mean ± SD	Intra P^1^	P^2^
IL-6, pg/ml	11.67 ± 10.84	13.72 ± 12.57	2.05 ± 11.77	0.935	8.15 ± 10.16	4.81 ± 5.08	−3.33 ± 6.41	0.011	0.034*
TNFα, pg/ml	30.04 ± 9.36	27.37 ± 9.78	−2.66 ± 8.31	0.223	25.57 ± 10.42	19.12 ± 6.82	−6.44 ± 7.21	< 0.0001	0.187
GSH, mM/l ^c^	202.72 ± 40.66	185.86 ± 42.72	−16.86 ± 45.06	0.094	176.70 ± 34.6	185.79 ± 45.74	9.08 ± 54.28	< 0.0001	0.513
MDA, μM/L	2.90 ± 0.47	2.80 ± 0.41	−0.13 ± 0.54	0.241	2.81 ± 0.48	2.69 ± 0.40	−0.12 ± 0.62	0.354	0.945
Albumin, g/dl	4.15 ± 0.37	4.24 ± 0.38	0.09 ± 0.38	0.282	4.15 ± 0.27	4.41 ± 0.37	0.25 ± 0.40	0.006	0.178*
Vitamin C, μM/L ^d^	27.00 ± 3.30	29.77 ± 6.13	2.77 ± 6.22	0.049	29.45 ± 4.47	35.45 ± 5.29	6.00 ± 6.71	< 0.0001	0.004*
Magnesium, mg/dl	1.98 ± 0.16	2.16 ± 0.14	0.17 ± 0.20	0.002	2.02 ± 0.16	2.13 ± 0.16	0.10 ± 0.25	0.054	0.754
Weight, kg	59.16 ± 7.66	59.80 ± 8.62	0.64 ± 2.68	0.263	59.92 ± 9.88	60.45 ± 9.79	0.52 ± 2.19	0.251	0.728
BMI, kg/m^2^	21.53 ± 2.59	21.73 ± 2.74	0.20 ± 0.97	0.338	20.65 ± 3.49	20.82 ± 3.40	0.17 ± 0.75	0.267	0.816
Calf circumference, cm	31.50 ± 2.01	31.71 ± 2.36	0.21 ± 1.29	0.430	31.41 ± 2.98	31.79 ± 2.77	0.37 ± 1.14	0.122	0.676
Arm circumference, cm	28.96 ± 2.66	29.0 ± 2.76	0.03 ± 1.01	0.855	28.60 ± 3.01	28.42 ± 2.98	−0.18 ± 1.43	0.538	0.592*
Lean body mass, kg	6.72 ± 0.46	6.18 ± 0.67	0.09 ± 0.38	0.248	6.98 ± 0.81	7.25 ± 1.03	0.26 ± 0.85	0.061	0.094*
Protein BIA, kg ^a^	8.91 ± 0.97	9.05 ± 1.2	0.13 ± 0.57	0.256	9.64 ± 1.27	10.25 ± 1.56	0.61 ± 1.03	0.008	0.019*
Fat-free mass, kg ^b^	45.35 ± 4.94	46.13 ± 5.99	0.78 ± 2.62	0.167	48.87 ± 6.17	51.72 ± 7.54	2.85 ± 4.65	0.006	0.025*
FFMI, kg/m^2^	16.41 ± 0.89	16.71 ± 1.27	0.29 ± 0.97	0.160	16.79 ± 1.89	17.78 ± 2.35	0.99 ± 1.61	0.006	0.018*
Right HGS, kg	23.35 ± 6.61	23.56 ± 7.66	0.20 ± 4.54	0.935	23.77 ± 6.58	26.53 ± 5.94	2.76 ± 3.81	0.003	**0.023**
Left HGS, kg	21.7 ± 7.33	21.99 ± 7.87	0.28 ± 4.32	0.795	23.38 ± 8.24	26.73 ± 7.29	3.34 ± 5.65	0.006	0.045*
FEV1, %	44.95 ± 14.16	44.34 ± 16.53	−0.60 ± 13.33	0.829	42.58 ± 16.74	45.70 ± 20.22	3.12 ± 8.37	0.081	0.218
FVC, %	60.54 ± 10.34	59.63 ± 11.66	−0.9 ± 10.45	0.601	59.29 ± 10.83	61.08 ± 14.32	1.79 ± 10.65	0.513	0.391
SGA score	10.43 ± 4.81	8.65 ± 2.82	−1.78 ± 4.43	0.067	10.37 ± 3.25	8.16 ± 2.11	−2.20 ± 3.20	0.003	0.899
SGRQ, total score ^e^	59.0 ± 14.0	58.0 ± 13.0	−1.0 ± 11.0	0.644	67.0 ± 13.0	55.0 ± 13.0	−12.0 ± 12.0	< 0.0001	0.040*
SGRQ, symptom score ^f^	63.0 ± 18.0	58.0 ± 16.0	−4.0 ± 17.0	0.245	73.0 ± 10.0	60.0 ± 12.0	−13.0 ± 17.0	0.001	0.575
SGRQ, activity score	65.0 ± 20.0	67.0 ± 23.0	1.0 ± 17.0	0.681	71.0 ± 19.0	67.0 ± 19.0	−4.0 ± 17.0	0.184	0.289
SQRQ, impact score ^g^	53.0 ± 16.0	52.0 ± 13.0	−1.0 ± 13.0	0.695	63.0 ± 14.0	46.0 ± 13.0	−17.0 ± 14.0	< 0.0001	0.021*
IADL score	5.04 ± 1.39	4.66 ± 1.74	−0.28 ± 0.90	0.161	5.50 ± 0.93	5.12 ± 1.19	−0.37 ± 1.13	0.131	0.965*
Katz index	5.56 ± 0.89	5.50 ± 0.91	−0.04 ± 0.48	0.655	5.45 ± 0.93	5.70 ± 0.85	0.25 ± 0.53	0.034	0.164*

Abbreviations: *BMI* Body mass index, *FFMI* Fat-free mass index, *HGS* Handgrip strength, *GSH* Glutathione, *TNFα* Tumor necrosis factor, *IL-6* Interlukin-6, *MDA* Malondialdehyde, *FEV*1 Forced expiratory volume in 1 s, *SGA* Subjective global assessment, *SGRQ* St. George’s respiratory questionnaire, *IADL* Instrumental activities of daily living

^1^ *P* values denote significance of within-group changes. ^2^ P values denote significance of between-group changes which were compared by ANCOVA (considering protein and magnesium intake as a fixed factor and baseline value as a covariate which was mentioned as ^a,b,c,d,e,f,g^

*Non-parametric were used for variables (Wilcoxon test and Mann-Whitney test were used to evaluate the within-group and between-group changes, respectively)

Whole fat-free mass, FFMI, and body protein were significantly increased in the intervention group compared to the control group (*P* = 0.025, *P* = 0.018 and *P* = 0.019, respectively). Handgrip strength results showed significant improvements in both hands in the intervention group compared to the control group (*P* = 0.023 and *P* = 0.045).

Assessment of malnutrition status indicated that serum albumin concentration significantly increased in the intervention group compared to the baseline. Moreover, there was a significant decrease in SGA score only in the intervention group. However, there were no statistically significant differences between two groups.

Serum vitamin C concentration significantly increased in the intervention group compared with the control group (*P* = 0.004). Also, plasma magnesium level increased in the control group, though no significant difference was observed between the two groups. Serum magnesium concentration significantly increased in the control group, while it had no changes in the intervention group during the study phase. On the other hand, changes in serum magnesium level were not significantly different between groups.

Evaluation of quality of life, regarding the respiratory function, illustrated a significant improve in SGRQ score and Katz scale in the intervention group. Finally, there were significant differences in total SGRQ score and the impact subscale of SGRQ between two groups (*P* = 0.040 and *P* = 0.021, respectively).

## Discussion

The present single-blind randomized controlled trial demonstrated novel findings on the clinical benefits of whey beverage fortified with magnesium and vitamin C in patients with moderate-to-severe COPD. There was a significant decrease observed over time in IL-6 as an inflammatory marker, and ultimately improvement in health-related quality of life in the intervention group, compared to the control group. This targeted nutritional support induced a dramatic increase in the whole fat-free mass, FFMI, and muscle strength. To our knowledge, this is the first study investigating the efficacy of consumption of whey beverage fortified with magnesium and vitamin C as a novel complex and targeted nutritional support in improving health status of patients with COPD.

Reducing systemic inflammation is one of the major goals in improving prognosis of advanced COPD. Systemic inflammation related to COPD is considered to aggregate the COPD complications [24]. It is interesting to note that there was a significant decrease in inflammatory cytokine levels in this study. The anti-inflammatory effects of our targeted dietary intervention were mainly due to both whey protein and magnesium considered as anti-inflammatory agents [11, 25–27]. In confirmation of our findings, recent trials have indicated that whey protein, as well as magnesium supplementation, decreased high blood inflammatory cytokine levels (IL-6 and TNF-a) through inhibiting the production and reducing the circulation of inflammatory cytokines [11, 25–27].

Cachexia is well known as a common and partially reversible aspect of COPD contributing to skeletal muscle wasting, reducing muscle strength, decreasing exercise capacity, and increasing mortality, independent of airflow limitation in patients with COPD [28]. The findings of this study revealed that consumption of whey beverage fortified with magnesium and vitamin C for 8 weeks had significant effects on the features of cachexia, including muscle depletion, reduced function, and systemic inflammation.

There is direct relationship between skeletal muscle dysfunction and progressive loss of muscle mass in patients with COPD [29]. There was an increase in whole fat-free mass (2.85 ± 4.65 kg), fat-free mass index, and total body protein content in the intervention group, which was greater than the control group. These changes might be related to the unique combination of whey protein and magnesium. The quantity and quality of dietary protein in order to increase protein anabolism and maintain muscle mass in the older adults and/or cachexic patients have been topic of interest in recent nutritional intervention researches [30, 31]. Whey protein, as a high-quality protein is shown to stimulate the net whole body protein gained by up-regulation of anabolic mTOR pathways (mammalian target of rapamycin pathways) with a particular role for the amino acid leucine. The beneficial effects of whey protein supplementation on muscle mass and strength in patients with COPD and older sarcopenic adults have been reported in similar findings, which is in agreement with our results [32, 33]. On the other hand, in Calder et al. study, nutrition intervention containing high doses of omega-3, vitamin D, and whey protein couldn’t improve fat-free mass significantly, in the cachexic COPD patients [31]. The possible explanation for this contradictory result might be related to positive effects of whey protein and magnesium interaction on fat-free mass.

Positive findings of the association of dietary magnesium with muscle mass and FFMI are reported in a number of cross-sectional studies [34–36]. Dietary magnesium plays a crucial role in metabolic, neuromuscular, and anti-inflammatory functions that might delay the loss of skeletal muscle mass and strength related to age [12]. Any change in magnesium status might be a risk factor for muscle depletion, because skeletal muscle mass is the largest store of magnesium in the body [37, 38]. Lack of change in magnesium concentration in the intervention group might be due to the retention of magnesium in the muscle according to the anabolic process happened in the patients regarding increases in fat-free mass component in our study. Moreover, there was no significant difference in serum magnesium concentrations between two groups. This finding might be due to our measurement method, because serum magnesium is not an accurate indicator [39] (because of poor health status of participants, it was impossible to measure magnesium concentrations in erythrocytes or urine).

Handgrip strength is known as a validated functional measure for nutritional status, skeletal muscle mass, and physical performance in the older adults and cachexic patients [40–43]. There has been an inverse relationship between handgrip strength and mortality in patients with COPD in recent studies [44–46]. In this trial, consumption of fortified whey beverage was effective in increasing handgrip strength. This result is in agreement with previous studies indicating positive effects of whey protein and magnesium supplementation on muscle strength.

Dietary antioxidants protect from smoke-induced oxidative stress and improve pulmonary function in patients with COPD [47]. The protective effect of dietary high intake of vitamin C on lung tissue might increase FEV1 and FVC as indicated in recent studies [16]. There was no significant difference in FEV1 over the time, while there were significant changes in serum vitamin C levels, and it is not in agreement with previous studies. This apparent lack of correlation might be referred to the dose-effect relationship between vitamin C intake and lung function [48]. Vitamin C high content was not supplied by our fortified beverage. Furthermore in recent evidence, whey protein has also been proposed as an antioxidant agent and it might stimulate the synthesis of glutathione as an intracellular antioxidant due to the specific role of the amino acid cysteine [49, 50]. The current study showed an increase in blood glutathione in the intervention group, while the difference between two groups was not significant. A possible explanation for lack of glutathione change might be that changes in inflammatory cells and tissues might not be depicted by whole blood values [51].

Considerable insight into improving health-related quality of life has been provided in our study, regarding the respiratory function measured by the SGRQ. There was a strong correlation between impaired HRQoL and severity of respiratory disease in patients with COPD [52–54]. The impact of daily consumption of whey beverage fortified with magnesium and vitamin C on COPD-related quality of life by improving psychological functioning and respiratory symptoms was generally remarkable. Based on available evidence, psychological improvements might be related to the decreased inflammatory cytokines [55] and neuroprotective effect of magnesium [56]. Our findings appear to be well supported by these trials indicating desirable effects on COPD comorbidities.

The unique composition of nutritional intervention, comprehensive patient evaluation, low drop-out rate, and high treatment compliance were the strength points of our study. However, we are aware that there are some limitations in our research. Participants did not receive placebo. Our study was only implemented on men, as there were few female patients among the eligible ones, and we do not know whether the results will be the same for women.

## Conclusions

Briefly, the evidence of this study implied that whey beverage fortified with magnesium and vitamin C could decrease inflammatory cytokine levels, improve indices of skeletal muscle mass and strength, and ultimately increase HRQoL in patients with moderate-to-severe COPD as a novel nutritional intervention. These effects could be due to the anabolic and anti-inflammatory effects of whey protein and magnesium. Unlike many studies stating that nutritional supplementation only might not be effective but its combination with exercise training is a beneficial approach, our study highlighted the possible effect of appropriate nutritional support independent of training in the patients with moderate-to-severe COPD. Therefore, future studies are suggested to better elucidate the best nutritional strategies for managing or improving the respiratory function and nutritional status in patients with COPD.
